# Exchange rate response to economic policy uncertainty: evidence beyond asymmetry

**DOI:** 10.1057/s41599-022-01372-5

**Published:** 2022-10-10

**Authors:** Bisharat Hussain Chang, Omer Faruk Derindag, Nuri Hacievliyagil, Mehmet Canakci

**Affiliations:** 1 Department of Business Administration, Sukkur IBA University, Sukkur, Pakistan; 2 Department of International Trade and Business, Inonu University, Battalgazi, Malatya, Turkey; 3 Department of Public Finance, Inonu University, Battalgazi, Malatya, Turkey

**Keywords:** Economics, Economics

## Abstract

Recent studies have examined the relationship between economic policy uncertainty and exchange rate. We contribute to this literature by considering the effect of minor positive and major positive changes as well as minor negative and major negative changes in the economic policy uncertainties on the exchange rates. In this regard, we use a recently developed multiple asymmetric threshold nonlinear ARDL model along with Granger causality in quantile test. Our estimates support the asymmetric effect in three countries only when an asymmetric ARDL model is used. However, these estimates support asymmetric effects for all the sample countries when the multiple asymmetric threshold nonlinear ARDL model is used. Moreover, the effect varies across various quantiles when Granger causality in quantile test is used. Overall, the extended model helps us to examine more minutely the impact of EPU and GEPU on the exchange rate in G7 countries. The results of this study can be useful for the central banks to devise appropriate policies to intervene in the foreign exchange market.

## Introduction

Uncertainty plays a negative role in economic activity. For instance, during uncertainty, firms do not make investment decisions until more information arrives in the future because the cost of investment decisions is irreversible (Bernanke, 1983). Moreover, economic policy uncertainty (EPU)^1^ affects international trade, economic sanctions, and macroeconomic variables. Therefore, following the seminal work by Bloom (2009), quantifying the effect of EPU on the aggregate economy has gained momentum among policymakers, practitioners, and academic scholars.

Various studies have examined the relationship between EPU and other economic and financial variables. One stream of the literature investigates the relationship between EPU and macroeconomic variables such as inflation, consumption, investments, economic development, money demand, unemployment, and financial distress (Hashmi and Chang, 2021; Hashmi et al., 2021; Jones and Olson, 2013; Brogaard and Detzel, 2015; Aastveit et al., 2017; Caggiano et al., 2017). In contrast, another stream of literature examines the impact of EPU on different asset classes such as commodities, derivatives and insurance, gold futures, bonds, and stocks (Syed et al., 2019; Hashmi et al., 2021; Hashmi et al., 2022; Arouri et al., 2016; Li et al., 2018; Fang et al., 2018; Uche et al., 2022b).

The above studies have examined the relationship between EPU, macroeconomic variables, and other asset classes. However, limited literature mainly focuses on the relationship between EPU and exchange rate. There are various ways through which EPU affects the exchange rate. First, Governments and other relevant policymakers devise policies regarding FDI and other macroeconomic indicators. However, uncertainties in these policies can prevent international investors from investing in a foreign country, affecting both inward and outward FDI. Since the payments against FDI are made in foreign currencies, changes in FDI and FDI-related policies, in turn, affect the exchange rates. Second, uncertainties in economic policies can affect exports and imports of a country, which changes the demand for a foreign currency; hence, it also changes the exchange rates. Similarly, changes in policies regarding interest rates also affect the borrowers in deciding whether to borrow in domestic or foreign currency; hence it affects the exchange rate.

However, despite the above theoretical link between EPU and exchange rate, limited empirical literature exists that examines the relationship among the underlying variables. Krol (2014) examined the effect of general economic uncertainty and economic policy uncertainty on the exchange rate volatility in ten developed and emerging economies. The author found that the general economic uncertainty has a more negligible impact on exchange rate volatility than the economic policy uncertainty. The author further concluded that in the developed economies, which are more integrated with the US economy, both domestic EPU and the US-EPU shocks affect the exchange rate. Whereas, in the developing economies, which are less integrated with the US, only domestic EPU affects the exchange rate.

Moreover, Kido (2016) used the DCC-GARCH model on monthly data to investigate the spillover effect of US economic policy uncertainty on exchange rates and concluded a negative and statistically significant correlation between US-EPU and high-yield currencies in different countries, except the Japanese Yen. Using factor-augmented vector auto-regression, Kido (2018) investigated the US-EPU impact on the Asian and global financial markets and found that an increase in US-EPU has a spillover effect on the commodity prices, exchange rates, and equity prices. He also concluded that an increase in the US-EPU causes an appreciation in Japanese Yen, whereas it causes depreciation in most of the other currencies. His findings further concluded an insignificant effect of US-EPU on the Chinese equity market.

The existing literature further mentions that macroeconomic variables exhibit nonlinearity over time. Several studies have been conducted that highlight the importance of nonlinear modeling. Lee and Lin (2012) argued that many macroeconomic variables show structural breaks over time and reflect nonlinear patterns in data series. Naifar and Al Dohaiman (2013) also mentioned that existing linear models do not capture the nonlinearity in the data series. Besides, Bildirici and Turkmen (2015) concluded that the explanatory power of a nonlinear model is more significant than linear models. Following these arguments, Makinayeri (2019) examined the nonlinear relationship between EPU and macroeconomic activity using the nonlinear ARDL model in G7 countries. His findings concluded that EPU asymmetrically affects the economic variables in G7 countries. Recent literature further examined the nonlinear relationship between EPU and exchange rate. For example, Yin et al. (2017) explored the causal relationship between EPU and exchange rate by applying the quantile regression test. Their findings indicated a more significant relationship when quantile regression is used. Moreover, Chen et al. (2019) also used quantile regression to investigate the effect of EPU on the exchange rate in China and concluded a heterogeneous impact of EPU on the exchange rate in China.

There are a few other recent studies as well which have been conducted to examine the relationship between economic policy uncertainty (EPU) and exchange rate. For example, Bartsch (2019) used GARCH models based on the daily frequency data and concluded that EPU has a stronger effect on the exchange rate when daily data is used. Using the nonlinear ARDL model, Kisswani and Elian (2021) examined the effect of oil prices, economic policy uncertainty, and geopolitical risk on the exchange rate volatility in the UK, Republic of Korea, Japan, China, and Canada. Their findings conclude that these variables significantly affect the exchange rate in some countries whereas insignificantly affect in other countries. Sohag et al. (2022) also conducted a study in Russia using quantile-based time series techniques. Their findings conclude that an increase in Russian economic policy uncertainty causes an appreciation in the local currency during managed floating exchange rates, whereas it causes a depreciation in the local currency during the floating exchange rate system. Similarly, Song et al. (2022) examined the network correlations between categorical economic policy uncertainties, exchange rates, and commodities in China. They concluded that USD/CHY mainly dominates China’s domestic system. Moreover, monetary policy uncertainty and fiscal policy uncertainty dominate China’s commodity returns.

The limitation of the above literature is that it does not differentiate the effect of positive and negative changes in the EPU on the exchange rate. More specifically, it does not distinguish the impact of minor to major positive shocks in the EPU and minor to major adverse shocks in the EPU on the exchange rates. However, investors demand more risk premium for bearing additional risk. Therefore, the investors and other stakeholders react more abruptly to negative news, such as increasing policy uncertainty, than positive news. In other words, foreign investors will make quick adjustments in their investment decisions when there is rising policy uncertainty in a foreign country compared to a stable policy. Moreover, since the change in the investment and policy decisions involve cost, investors may not change their choices during minor policy changes. In contrast, they may change decisions when there are significant changes in the foreign policy uncertainties.

This study extends the literature by using extended methodologies such as a recently developed multiple asymmetric threshold nonlinear ARDL (MATMARDL) model proposed by Uche et al. (2022a). The MATNARDL model combines both NARDL (Shin et al., 2014) and MTNARDL model (Pal and Mitra, 2015, 2016). The advantage of the MATNARDL model is that it considers the effect of minimal and extensive adverse shocks and minimal and extensive positive shocks in the explanatory variable on the explained variable, which the previous models fail to examine. However, this model captures the effect across various shocks of the explanatory variable only. Therefore, to consider the impact across quantiles of the explanatory and explained variables, we use the Granger causality in quantile (GCQ) test, which has not been used in the given context. Finally, this study compares the results of the MATNARDL model with the standard NARDL model.

Our study contributes to the existing literature in various ways: First, using a nonlinear ARDL cointegration modeling approach, this study examines the asymmetric impact of domestic EPU on the exchange rates in G7 countries. Second, since foreign shocks also influence the domestic exchange rate, we also look at the effect of global EPU (GEPU) on the exchange rate. Finally, we differentiate the impact of minor to major positive shocks in EPU and GEPU and the effect of minor to major negative shocks in EPU and GEPU on the exchange rates in G7 countries.^2^

The present study explores the effect of domestic EPU and Global EPU (GEPU) on the exchange rate in G7 countries. The selection of the G7 countries as a venue for research is based on the following reasons. First, The G7 countries’ sample is the most industrialized and represents 58% (i.e., $ 317 trillion) of the Global net worth (OECD, 2019). Second, these sample countries represent more than 46 and 32% of global gross domestic products based on nominal price and purchasing power parity, respectively (IMF, 2018). Moreover, the economic performance of these countries has significantly improved over time. Also, this block (i.e., G7) spends billions of dollars on research and development, which has enhanced these countries (Yuan et al., 2021). The unique characteristics of these countries and the largest share of global net worth motivates us to explore the effect of EPU and GEPU on the exchange rate in G7 countries.

The remainder of the paper is structured as follows: Section “Data and methodology” describes the data and explains the methods to carry out this study, section “Empirical results” presents the empirical findings, and section “Conclusion, policy implications and limitations of the study” concludes the research and proposes policy recommendations.

## Data and methodology

### Data overview

This study investigates whether the effect of EPU and global EPU (GEPU) on exchange rate varies from minor to major positive shocks in the EPU and GEPU and from minor to major adverse shocks in the EPU and GEPU in G7 countries. To do this, we use monthly data from January 1998 to January 2021. The sample period is selected based on the data availability of the variables under study. The current study uses monthly data to contain more information than quarterly data. Besides, monthly data is less noisy than the daily data series (Driesprong et al., 2008). The G7 countries include Canada, France, Germany, Japan, the United Kingdom (UK), and the United States (US). The dependent variable is the real effective exchange rate (REER), which measures the value of a local currency against the basket of foreign currencies. The primary independent variable is economic policy uncertainty (EPU) where this index is developed by Baker et al. (2016). This is news-based index that is used to measure the uncertainties of the economic policies developed by relevant countries. Moreover, the industrial production index (IPI) and consumer price index (CPI) are used as control variables. Finally, the GEPU variable is also taken to see its impact on the exchange rate for a robustness purpose. The data of all the variables, except EPU and GEPU, are obtained from International Financial Statistics (IFS), an International Monetary Fund (IMF) database. The data for EPU and GEPU, adjusted for purchasing power parity (GEPU-PPP), is extracted from the Baker et al. (2016) webpage^3^. All the data series are transformed into natural logarithms that interpret the coefficients as elasticity.

### Methodologies

The primary objective of this study is to examine the impact of substantial and minimal changes in the EPU and global EPU (GEPU) on the real effective exchange rate in G7 countries. For this purpose, we use the nonlinear ARDL (NARDL) model proposed by Shin et al. (2014) and the MATNARDL model recently proposed by Uche et al. (2022a). Moreover, we also use the Granger causality in quantile (GCQ) test to analyze causality across different quantiles.

#### Asymmetric ARDL model

In this study, the NARDL model helps differentiate the effect of positive and negative changes in the EPU and GEPU on the exchange rate. In contrast, the MATNARDL model helps determine the impact of minor to major positive shocks in the EPU and GEPU and the effect of minor to major adverse shocks in the EPU and GEPU on the exchange rate. As the NARDL model is the extended version of the ARDL model, we present the general form of unrestricted error correction of the ARDL bounds testing approach. This approach is based on the assumption that the dependent variable responds in a similar (linear) way to both positive (increase) and negative (decrease) shocks to the explanatory variables. The general form of the standard linear ARDL model is expressed as follows:

1 $$\Delta Y_t = \mu + \rho Y_{t - 1} + \theta X_{t - 1} + \mathop {\sum}\limits_{J = 1}^{p - 1} {\alpha _J\Delta Y_{t - J}} + \mathop {\sum}\limits_{J = 0}^{q - 1} {\beta _J\Delta X_{t - J}} + \varepsilon _t$$

where Δ denotes the difference operator,$Y_t$ represents the dependent variable, *μ* shows intercept, *X*_*t*_ shows all the independent variables in Kx1 vector form. The long-run coefficients are represented by *ρ* and *θ*, whereas the short-run coefficients are represented by *α* and *β*. The lag order of the dependent and independent variables is shown by *p* and *q*, respectively. Finally, $\varepsilon _t$ defines the error term. The null hypothesis of no cointegration for the ARDL model is tested (*ρ* = *θ* = 0) against the alternative hypothesis of cointegration ($\rho \,\ne\, \theta \,\ne$ 0). We use the *F*-test proposed by Pesaran et al. (2001) to test the null hypothesis. The *F*-test calculates the lower and upper bounds critical values at any given significance level. We reject the null hypothesis if the test value is above upper bounds critical values. In contrast, we do not reject the null hypothesis if it falls below the lower bounds of critical values. Finally, the inference would remain inconclusive if the value falls in between the lower and upper bounds critical values.

The primary assumption of the above ARDL model is that all independent variables have symmetric effects on the dependent variable. However, in real life, this may not be the case. Shin et al. (2014) proposed a nonlinear ARDL model (NARDL), which assumes that the relationship between independent and dependent variables is asymmetric. This model captures the asymmetric effect both in the long- and short-run by decomposing the independent variables into a partial sum of positive and negative shocks. In this study, we decompose the EPU into positive and negative surprises in the following manner:

2a $${\mathrm{EPU}}_t^ + = \mathop {\sum}\limits_{j = 1}^t {\Delta {\mathrm{EPU}}_j^ + } = \mathop {\sum}\limits_{j = 1}^t {{\mathrm{max}}\left( {\Delta {\mathrm{EPU}}_j,0} \right)}$$

2b $${\mathrm{EPU}}_t^ - = \mathop {\sum}\limits_{j = 1}^t {\Delta {\mathrm{EPU}}_j^ - } = \mathop {\sum}\limits_{j = 1}^t {{\mathrm{min}}\left( {\Delta {\mathrm{EPU}}_j,0} \right)}$$

Using Eqs. 2a and 2b, the asymmetric NARDL model can be expressed as follows:

3 $$\begin{array}{l}\Delta {\mathrm{lnREER}}_t = \alpha _0 + \alpha _1{\mathrm{lnREER}}_{t - 1} + \alpha _2^ + {\mathrm{lnEPU}}_{t - 1}^ + + \alpha _2^ - {\mathrm{lnEPU}}_{t - 1}^ - + \alpha _3{\mathrm{lnIPI}}_{t - 1}\\ \quad\quad\quad\quad\quad\quad+ \,\alpha _4{\mathrm{lnCPI}}_{t - 1} + \mathop {\sum}\limits_{i = 1}^p {\gamma _{5,i}\Delta {\mathrm{lnREER}}_{t - i}} + \mathop {\sum}\limits_{i = 0}^q {\left( {\gamma _{6.i}^ + \Delta {\mathrm{lnEPU}}_{t - i}^ + + \gamma _{6.i}^ - \Delta {\mathrm{lnEPU}}_{t - i}^ - } \right)} \\ \quad\quad\quad\quad\quad\quad + \mathop {\sum}\limits_{i = 0}^r {\gamma _{7,i}\Delta {\mathrm{lnIPI}}_{t - i}} + \mathop {\sum}\limits_{i = 0}^s {\gamma _{8,i}\Delta {\mathrm{lnCPI}}_{t - i}} + \varepsilon _t\end{array}$$

where ln indicates that all variables have been used in a natural logarithm, whereas *p*, *q*, *r*, and *s* represent the lag order against each variable in the short run, the long-run asymmetry is examined using Wald-test under the null hypothesis: $a_2^ + = a_2^ -$. The rejection of the null hypothesis would confirm an asymmetric relationship in the long run. Next, to find the short-run asymmetry, we apply Wald-test under the null hypotheses: $\gamma _{6{{{\mathrm{i}}}}}^ + = \gamma _{6{{{\mathrm{i}}}}}^ -$. The rejection of the null hypothesis, in this case, would confirm the asymmetric relationship in the short run. Finally, we apply the *F*-test and the Wald-test for the joint cointegration test. The null hypothesis under this test is that all the long-run coefficients are jointly equal to zero.

#### Multiple asymmetric thresholds ARDL (MATNARDL) model

Pal and Mitra (2015, 2016) introduced the multiple threshold nonlinear ARDL model, motivated by the nonlinear NARDL model (Shin et al., 2014), which does not consider substantial and minimal changes in the exogenous variable on the dependent variable. However, it can only capture the impact of the partial sum of positive and negative shocks in the exogenous variable. On the other hand, the multiple thresholds nonlinear ARDL (MTNARDL) model, proposed by Pal and Mitra (2015, 2016), considers the effect of substantial and minimal changes in the exogenous variable on the dependent variable. Uche et al. (2022a) further extended the MTNARDL model by dividing positive and negative shocks into multiple thresholds and naming this extended model multiple asymmetric thresholds nonlinear ARDL (MATNARDL) model. We use this extended model in our case. It helps us examine the effect of minor to major adverse shocks and minor to major positive surprises in the explanatory variable on the explained variable.

Therefore, the present study uses this advanced model to understand the comprehensive relationship between EPU and exchange rate in G7 countries. In this regard, the EPU variable is decomposed into three positive and three adverse shocks series as follows:

4a $${\mathrm{EPU}}_t^ - = {\mathrm{EPU}}_o^ - + {\mathrm{EPU}}_t^ - \left( {\omega _1} \right) + {\mathrm{EPU}}_t^ - \left( {\omega _2} \right) + {\mathrm{EPU}}_t^ - \left( {\omega _3} \right)$$

4b $${\mathrm{EPU}}_t^ + = {\mathrm{EPU}}_o^ + + {\mathrm{EPU}}_t^ + \left( {\omega _1} \right) + {\mathrm{EPU}}_t^ + \left( {\omega _2} \right) + {\mathrm{EPU}}_t^ + \left( {\omega _3} \right)$$

where ${\mathrm{EPU}}_t^ - \left( {\omega _1} \right),$ ${\mathrm{EPU}}_t^ - \left( {\omega _2} \right)$, ${\mathrm{EPU}}_t^ - \left( {\omega _3} \right)$ in Eq. 4a are the three partial sum series of negative shocks in EPU set 30th and 70th thresholds, respectively. In contrast, ${\mathrm{EPU}}_t^ + \left( {\omega _1} \right),$ ${\mathrm{EPU}}_t^ + \left( {\omega _2} \right)$, ${\mathrm{EPU}}_t^ + \left( {\omega _3} \right)$ in Eq. 4b are the three partial sum series of positive shocks in EPU set 30th and 70th thresholds, respectively. These thresholds can be represented as ${\mathrm{EPU}}_t^ - \left( {\omega _1} \right),{\mathrm{EPU}}_t^ - \left( {\omega _2} \right),{{{\mathrm{and}}}}\,{\mathrm{EPU}}_t^ - (\omega _3)$ and ${\mathrm{EPU}}_t^ + \left( {\omega _1} \right),{\mathrm{EPU}}_t^ + \left( {\omega _2} \right),{{{\mathrm{and}}}}\,{\mathrm{EPU}}_t^ + \left( {\omega _3} \right)$ and are calculated as given below:

5a $${\mathrm{EPU}}_t^ - \left( {\omega _1} \right) = \mathop {\sum}\limits_{j = 1}^n {\Delta {\mathrm{EPU}}_t^ - \left( {\omega _1} \right)} = \mathop {\sum}\limits_{j = 1}^n {\Delta {\mathrm{EPU}}_j^ - I\left\{ {{{\Delta }}{\mathrm{EPU}}_j^ - \,<\, Q_{30}} \right\}}$$

5b $${\mathrm{EPU}}_t^ - \left( {\omega _2} \right) = \mathop {\sum}\limits_{j = 1}^n {\Delta {\mathrm{EPU}}_t^ - \left( {\omega _2} \right)} = \mathop {\sum}\limits_{j = 1}^n {\Delta {\mathrm{EPU}}_j^ - I\left\{ {Q_{30} \le \Delta {\mathrm{ER}}_j^ - \,\le\, Q_{70}} \right\}}$$

5c $${\mathrm{EPU}}_t^ - \left( {\omega _3} \right) = \mathop {\sum}\limits_{j = 1}^n {\Delta {\mathrm{EPU}}_t^ - \left( {\omega _3} \right)} = \mathop {\sum}\limits_{j = 1}^n {\Delta {\mathrm{EPU}}_j^ - I\left\{ {{{\Delta }}{\mathrm{EPU}}_j^ - \,>\, Q_{70}} \right\}}$$

6a $${\mathrm{EPU}}_t^ + \left( {\omega _1} \right) = \mathop {\sum}\limits_{j = 1}^n {\Delta {\mathrm{EPU}}_t^ + \left( {\omega _1} \right)} = \mathop {\sum}\limits_{j = 1}^n {\Delta {\mathrm{EPU}}_j^ + I\left\{ {{{\Delta }}{\mathrm{EPU}}_j^ + \,<\, Q_{30}} \right\}}$$

6b $${\mathrm{EPU}}_t^ + \left( {\omega _2} \right) = \mathop {\sum}\limits_{j = 1}^n {\Delta {\mathrm{EPU}}_t^ + \left( {\omega _2} \right)} = \mathop {\sum}\limits_{j = 1}^n {\Delta {\mathrm{EPU}}_j^ + I\left\{ {Q_{30} \le \Delta {\mathrm{ER}}_j^ + \,\le\, Q_{70}} \right\}}$$

6c $${\mathrm{EPU}}_t^ + \left( {\omega _3} \right) = \mathop {\sum}\limits_{j = 1}^n {\Delta {\mathrm{EPU}}_t^ + \left( {\omega _3} \right)} = \mathop {\sum}\limits_{j = 1}^n {\Delta {\mathrm{EPU}}_j^ + I\left\{ {{{\Delta }}{\mathrm{EPU}}_j^ + \,>\, Q_{70}} \right\}}$$

In the above Eqs. 5a, 5b, 5c, 6a, 6b, and 6c, *I*{*T*} represents the indicator function where its value is equal to 1 if the condition in parenthesis is satisfied or 0 otherwise. The decomposition of EPU into three negative partial sum series (5a, 5b, 5c) and three positive partial sum series (6a, 6b, 6c) can be expressed using the multiple asymmetric thresholds NARDL (MATNARDL) model proposed by Uche et al. (2022a), which is presented below:7where *k* = *j* + 3.

In equation 7, the null hypothesis of no long-run cointegration can be tested through : *ɖ*1 = *ɖ*2 = *ɖ*3 = *ɖ*4 = *ɖ*5 = *ɖ*6 = *ɖ*7 = *ɖ*8 = *ɖ*9 = 0. The critical values given by Pesaran et al. (2001) have been used to test the long-run cointegration. Moreover, the short-run asymmetry is tested using Wald-test test such as HO: ${{{\mathrm{\mu }}}}_{k1} = {{{\mathrm{\mu }}}}_{k2} = {{{\mathrm{\mu }}}}_{k3} = {{{\mathrm{\mu }}}}_{k4} = {{{\mathrm{\mu }}}}_{k5} + {{{\mathrm{\mu }}}}_{k6} = 0.$ Similarly the long-run asymmetry is tested using Wald-test for null hypothesis such as: HO: *ɖ*4 = *ɖ*5 = *ɖ*6 = *ɖ*7 = *ɖ*8 = *ɖ*9 = 0.

#### Granger causality in Quantiles test

We also use the Granger causality test in our analysis. Granger causality test is used to examine the causal relationship among the given variables. This test assumes that the dependent variable is explained independently of the lags of the independent variables. Researchers have extended the Granger causality test using advanced and diverse techniques. Our study uses the Granger causality test in quantiles proposed by Troster (2018) to examine the causality quantiles between EPU and exchange rate and between global EPU and exchange rates in G7 countries. Like the Granger causality test by Granger (1969), this test assumes that variable ${{{\mathrm{X}}}}_i$ does not Granger cause variable ${{{\mathrm{Y}}}}_i$ across different quantiles. This study believes that vector $\left( {P_i = P_i^X,P_i^y} \right)^\prime \in R^e,\,s = o + r,$ where $P_i^y$ indicates the preceding demonstration group of $\left( {{{P}}_iP_i^y = {{P}}_{i - 1, \ldots ..,}{{P}}_{i - r}} \right)^\prime$. Moreover, the null hypothesis of no causality under this test from ${{{\mathrm{Y}}}}_i\,to\,{{{\mathrm{X}}}}_i$ is represented as given below:

8 $$H_0^{y \to X}: = {{F}}_X\left( {P_i^X,P_i^y} \right) = {{F}}_X\left( {P_i^X} \right),\,{{{\mathrm{for}}}}\,{{{\mathrm{all}}}}\,x \in R,$$

where ${{F}}_X\left( {P_i^X,P_i^y} \right)$ indicates the interim distribution motive for variable ${{X}}_i$ that gives $\left( {P_i^X,P_i^y} \right)$. The null hypothesis in Eq. 8 conformed with Granger (1969). This study uses the ${{D}}_i$.T. for the QAR approach *m*(.) regarding all $\pi \in \Gamma \subset \left[ {0,1} \right]$. The null hypothesis under the causal non-Granger causality test is denoted as under:

9 $${\mathrm{QAR}}\left( 1 \right):m^1\left( {P_i^X,\partial \left( {\uppi} \right)} \right) = {\upgamma}_1\left( {\uppi} \right) + {\upgamma}_2\left( {\uppi} \right)Y_{i - 1} + \mu _t\delta _\sigma ^{ - 1}\left( {\uppi} \right)$$

In Eq. 9, the coefficient $\partial \left( {\uppi} \right) = {\upgamma}_1\left( {\uppi} \right),{\upgamma}_2\left( {\uppi} \right),{{{\mathrm{and}}}}\,\mu _t$ are approximately denoted using maximum probability based on the similar point of quantiles. Moreover, the reverse of a standard primary distribution function is represented by $\delta _\sigma ^{ - 1}\left( {\uppi} \right)$. To examine the causality, this study uses the QAR approach for Eq. 9 by simultaneously using the lagged to the alternative variables. Finally, the fundamental equation of the QAR (1), along with equation seven, is formulated as given below:

10 $$Q_{\uppi}^X\left( {P_i^X,P_i^y} \right) = {\upgamma}_1\left( {\uppi} \right) + {\upgamma}_2\left( {\uppi} \right)X_{i - 1} + \partial \left( {\uppi} \right)Y_{i - 1} + \mu _t\delta _\varepsilon ^{ - 1}\left( {\uppi} \right)$$

#### Diagnostic tests

Moreover, to examine the goodness of fit of the models and other requirements of the models used in this study, we use stability and other diagnostic tests. Ramsey RESET is used to examine whether the models are correctly specified, the serial correlation test is used to determine whether there is no autocorrelation issue in the models, and CUSUM and CUSUMQ tests are used to examine whether the models are stable. Finally, an adjusted *R*-square is used to determine whether the models are a good fit.

## Empirical results

### Descriptive statistics, unit root tests, and cointegration test

We report the descriptive statistics and other preliminary stationarity tests before conducting the primary analysis. Table 1 reports the descriptive statistics of the exchange rate, economic policy uncertainty (EPU), global EPU (GEPU), and consumer price index (CPI) for G7 countries. Findings indicate that most variables are positively skewed, reflecting non-symmetric distribution. Moreover, the kurtosis value is greater than 3, showing more peaked data with a flatter tail than the Gaussian distribution. Hence, the data series departs from the normality, which indicates the rejection of the null hypothesis of normal distribution. Jarque-Bera test also supports that data is not normally distributed.

**Table 1:** Descriptive statistics.

G7 Countries	Variables	MEAN	SD	Skewness	Kurtosis	JB
Canada	REER	87.081120	9.776309	0.14	1.73	18.65*
	EPU	154.6118	95.21873	0.99	3.46	45.77*
	IPI	109.5790	7.860737	0.83	4.84	68.44*
	CPI	97.70129	11.45384	−0.07	1.86	14.46*
France	REER	99.39121	4.561112	−0.04	1.69	19.02*
	EPU	168.1016	103.2053	0.75	3.53	28.50*
	IPI	104.9190	5.052865	0.04	1.95	12.05*
	CPI	97.19064	8.810852	−0.25	1.71	21.04*
Germany	REER	100.9888	5.245093	0.09	1.84	15.09*
	EPU	133.5567	64.43861	1.29	5.76	18.16*
	IPI	100.8502	9.961183	−0.20	1.61	22.94*
	CPI	98.13254	9.104176	−0.02	1.69	18.91*
Italy	REER	98.96674	3.877541	0.04	1.86	14.44*
	EPU	108.5982	38.15396	0.78	3.63	31.57*
	IPI	105.4566	10.31525	−0.01	1.36	29.40*
	CPI	96.57500	10.87722	−0.31	1.76	22.57*
Japan	REER	93.92185	15.92282	0.16	2.05	11.08*
	EPU	109.1315	35.41089	1.07	4.38	72.38*
	IPI	100.3186	6.265100	0.10	4.52	26.27*
	CPI	101.9386	1.802405	0.42	1.98	19.47*
UK	REER	114.0965	13.09729	−0.02	1.30	31.66*
	EPU	122.9583	69.62327	2.06	11.39	96.90*
	IPI	104.5190	5.351412	−0.07	1.45	26.65*
	CPI	97.74080	13.15200	0.14	159	22.73*
US	REER	110.0916	9.200616	−0.04	2.09	9.05*
	EPU	122.9997	47.77229	−0.98	3.84	50.38*
	IPI	104.9784	6.505803	−0.03	2.01	10.87*
	CPI	96.73891	13.03277	−0.15	1.75	18.14*
	GEPU	116.6500	51.94704	1.18	4.18	77.99*

Table 1 reports the descriptive statistics such as mean, standard deviation, and Skewness kurtosis Jarque_Berra (JB) test of all the G7 countries. The variables used in the study are: real effective exchange rate (REER), economic policy uncertainty (EPU), industrial production index (IPI), consumer price index (CPI), and global economic policy uncertainty (GEPU). JB test checks the data normality where the rejection of the null hypothesis indicates nonmorality of the data. * indicates the rejection of the null hypothesis at a 1% significance level.

Since NARDL and multiple asymmetric thresholds NARDL (Uche et al., 2022a) models require that variables be integrated of either order zero I(0) or I(1), we also use some preliminary tests for stationarity to ensure the order of integration of the variables. One of the advantages of the bounds testing approach is that it relaxes the assumption of the integrating order of the variables as either zero *I (0)* or one *I (1)*, or mixed order (Chang and Rajput, 2018 and Chang et al., 2018). However, the bounds testing approach can not be used when variables are integrated into order two *I(2)*. Therefore, to check the order of integrating the variables, we use standard unit root tests, such as augmented Dickey-Fuller (ADF) and Kwiatkowski PhillipsSchmidt Shin (KPSS) tests. The null hypothesis under the ADF test is that the series has a unit root, whereas the null hypothesis under KPSS test statistics shows that the series has no unit root. Table 2 shows the level and first difference results and ensures that none of the variables are at I (2). Hence we proceed further with the primary analysis.

**Table 2:** ADF and KPSS unit root tests.

G7 Countries	Variables	ADF test at level	ADD test at first difference	KPSS test at level	KPSS test at first difference
Canada	REER	−1.52	−13.01***	0.703***	0.17
	EPU	−1.36	−13.41***	1.15***	0.07
	IPI	−0.99	−7.33**	0.61***	0.18
	CPI	−1.99	−5.10***	2.12***	0.11
France	REER	−1.75	−12.91***	1.35***	0.05
	EPU	−1.56	−13.18***	0.49***	0.14
	IPI	−1.71	−22.36***	0.99***	0.04
	CPI	−1.92	−3.36***	2.10***	0.29
Germany	REER	−1.46	−12.26***	1.60***	0.03
	EPU	−1.2	−15.49***	1.68***	0.05
	IPI	−1.93	−6.00***	1.77***	0.05
	CPI	−1.00	−3.73***	2.13***	0.11
Italy	REER	−1.55	−6.59***	0.74***	0.17
	EPU	−1.896	−10.86***	1.557***	0.10
	IPI	−1.21	−4.63***	1.60***	0.06
	CPI	−2.74	−3.63**	2.08***	1.05
Japan	REER	−1.17	−10.84***	1.73***	0.18
	EPU	−5.56	−7.93***	1.59***	0.14
	IPI	−3.47	−9.53***	0.19***	0.02
	CPI	−1.08	−3.29***	0.60***	0.37
UK	REER	−3.59	−15.32***	0.35***	0.01
	EPU	−3.48	−10.74***	0.18***	0.16
	IPI	−1.26	−20.25***	1.54***	0.09
	CPI	0.02	−2.96***	2.14***	0.14
US	REER	−2.66	−12.79***	1.08***	0.06
	EPU	−2.57	−8.82***	0.70***	0.27
	IPI	−2.05	−4.19***	1.35***	0.05
	CPI	−1.91	−4.90***	2.11***	0.17
	GEPU	−2.01	−8.31***	1.37***	0.10

Table 2 presents the unit root test results using ADF and KPSS tests at the level and first difference of all the G7 countries. The variables used are real effective exchange rate (REER), economic policy uncertainty (EPU), industrial production index (IPI), consumer price index (CPI), and global economic policy uncertainty GEPU. The null hypothesis under the ADF test is that the series has a unit root, while KPSS null hypotheses have no unit root. The asterisk ** and *** indicate the rejection of the null hypothesis at a 5% and 1% significance level, respectively.

To test the null hypotheses of no cointegration, we use *F*-test proposed by Pesaran et al. (2001). This test examines the joint significance of the lagged variables in Eq. (3). Table 3 reports the *F*-statistic values for the bounds testing approach, where panels A and B present the results related to the NARDL and MATNARDL models, respectively. The NARDL bounds test estimates (Table 3-panel A) indicate that the null hypothesis of no cointegration is rejected in Canada, Japan, and the UK. These estimates, therefore, suggest long-run cointegration in Canada, Japan, and the UK.

**Table 3:** Bounds test results of NARDL and MATNARDL models with EPU series.

Panel A: NARDL model	Canada	France	Germany	Italy	Japan	UK	US
*F*-Statistic	4.211**	2.03	1.48	2.14	5.521***	7.711***	3.46*
Panel B: MATNARDL model							
*F*-Statistic	7.39***	8.01***	8.511***	12.01***	8.51***	7.60***	12.12***

Table 3 reports the results of the bounds testing approach for cointegration under the NARDL MATNARDL framework using monthly data of G7 countries. Consistent with Pesaran et al. (2001), we use the *F*-statistic values in panels A and B to test the null hypothesis of no cointegration between the exchange rate and economic policy uncertainty. ***, **, and * indicate the rejection of the null hypothesis of no cointegration at a 1%, 5% and 10% significance levels.

As mentioned earlier, the NARDL model does not consider the effect of minimal and substantial positive and negative shocks in the primary independent variable (i.e., EPU). Therefore, using the recently developed MATNARDL model by Uche et al. (2022a), we decompose the EPU into three positive partial sum series and three negative partial sum series. The positive partial sum series divides the positive shocks into major and minor changes, and the negative partial sum series divides the adverse shocks into major and minor changes. Therefore, the MATNARDL model helps differentiate the effect of minor to major positive changes in the EPU and minor to major negative changes in the EPU on the exchange rate in the G7 countries. Panel B (Table 3) presents the bounds test results for the MATNARDL model. These results indicate that long-run cointegration exists in all G7 countries, which, therefore, supports this model’s superiority over the standard NARDL model.

### NARDL estimation results

Additionally, we check the short- and long-run asymmetry between EPU and exchange rate using the Wald-test and present results in Table 4. The Wald-tests for NARDL asymmetry indicate that the null hypothesis of symmetry is rejected both in the short and long run for Canada and Japan, whereas it is rejected in the short run only for the UK. Moreover, for France, Germany, Italy, and the US, the null hypothesis is not rejected in the short and long run.

**Table 4:** Wald tests for short-and long-run symmetry with EPU series.

	Wald-test for NARDL estimates			Wald-test for MATNARDL estimates		
G7 Countries	Wald-test long-run	Wald-test short-run	Conclusion	Wald-test long-run	Wald-test short-run	Conclusion
Canada	6.4833*** [0.0115]	11.04573*** [0.0010]	Long-run and short-run asymmetry	9.6468*** [0.0005]	8.07836*** [0.00118]	Long-run and short-run asymmetry
France	0.310903 [0.5777]	0.411755 [0.5217]	Symmetry	8.8687*** [0.0012]	12.5739*** [0.003542]	Long-run and short-run asymmetry
Germany	0.568786 [0.4515]	0.188317 [0.6647]	Symmetry	8.1559*** [0.0032]	12.9219*** [0.005404]	Long-run and short-run asymmetry
Italy	2.02544 [0.1033]	2.712815 [0.1009]	Symmetry	7.8440*** [0.0009]	12.71599*** [0.00761]	Long-run and short-run asymmetry
Japan	8.5366*** [0.0023]	9.443981*** [0.005059]	Long-run and short-run asymmetry	12.6447*** [0.0015]	8.019164*** [0.00010]	Long-run and short-run asymmetry
UK	1.16852 [0.1423]	8.568018*** [0.00104]	Short-run aymmetry	9.745960*** [0.0012]	8.38712*** [0.0015]	Long-run and short-run asymmetry
US	1.6010 [0.4263]	1.549207 [0.2145]	Symmetry	12.2962*** [0.00011]	9.3871*** [0.0015]	Long-run and short-run asymmetry

Table 4 summarizes the long-run and short-run symmetry results for NARDL and MATNARDL models. For the NARDL model, the long-run symmetry is tested under the null hypothesis $\alpha _2^ +$= $\alpha _2^ -$. In contrast, short-run symmetry is tested under the null hypothesis $\gamma _{6,i}^ +$ = $\gamma _{6,i}^ -$. Likewise, for MATNARDL model the long-run symmetry is tested under the null hypothesis *ɖ*4 = *ɖ*5 = *ɖ*6 = *ɖ*7 = *ɖ*8 = *ɖ*9 = 0. In contrast, short-run symmetry for the MATNARDL model is tested under the null hypothesis ${{{\mathrm{\mu }}}}_{k1} = {{{\mathrm{\mu }}}}_{k2} = {{{\mathrm{\mu }}}}_{k3} = {{{\mathrm{\mu }}}}_{k4} = {{{\mathrm{\mu }}}}_{k5} + {{{\mathrm{\mu }}}}_{k6} = 0$. The corresponding *P*-values in the parenthesis test the null hypothesis of no symmetry. *** indicates the rejection of the null hypothesis at 1% significance level.

Next, we present the NARDL estimates in Table 5, where panels A, B, and C indicate the short-run, long-run, and diagnostic tests. For optimal lag selection, we use Akaike information criteria (AIC). Panel A shows the partial decomposition of EPU shocks into positive (increase) and negative (decrease) coefficients and denoted as $\Delta {\mathrm{EPU}}^ +$ and $\Delta {\mathrm{EPU}}^ -$. These short-run coefficients differ in terms of the sign and size; when the EPU shock is positive (e.g., $\Delta {\mathrm{EPU}}^ +$), it negatively and significantly affects the exchange rate in Canada, Japan, and the UK. In contrast, when the EPU shock is negative (e.g., $\Delta {\mathrm{EPU}}^ +$), it does not significantly affect the exchange rate in these countries. Therefore, these results suggest possible short-run asymmetry in Canada, Japan, and the UK. On the contrary, both positive and negative shocks in EPU insignificantly affect the exchange rates in the short-run in France, Germany, Italy, and the US.

**Table 5:** NARDL estimates with EPU series.

G7 countries	Canada	France	Germany	Italy	Japan	UK	US
Panel A: Short-run coefficients							
$\Delta {\mathrm{REER}}\,( - 1)$	0.206***	0.206***	0.261***	0.241***	0.271***	0.412	0.372***
$\Delta {\mathrm{REER}}\,( - 2)$	0.212	0.124	0.235	0.254	0.147	0.251	−0.131**
ΔEPU^+^	−0.020***	−0.002	−0.002	0.0006	0.051***	−0.016***	0.012
ΔEPU^+^ (−1)	0.212***	0.251	0.254	0.124	−0.049***	0.012**	−0.003
ΔEPU^+^ (−2)	0.234	0.152	0.184	0.235	0.027**	0.124	0.415
ΔEPU^–^	−0.004	0.002	−0.002	0.001	−0.008	0.124	0.254
ΔEPU^–^ (−1)	0.124	−0.004	−0.005	0.124	0.034**	0.147	0.251
ΔEPU^–^ (−2)	0.124	0.014	0.110	0.135	−0.029***	0.123	0.235
ΔIPI	−0.034**	0.019*	−0.002	0.017*	0.049	0.263***	0.140
ΔIPI (–1)	0.124	0.014	0.142	0.254	0.245***	−0.209	−0.071***
ΔIPI (–2)	0.157	0.124	0.147	0.174	−0.255***	0.303***	−0.361***
ΔCPI	0.984***	0.288**	0.501***	0.584	0.147	0.088	−0.165***
ΔCPI (–1)	−1.029***	0.124	0.184	−0.635***	0.125	0.125	0.235
ΔCPI (–2)	0.241	0.142	0.124	0.325	0.142**	0.241	0.241
Panel B: Long-run coefficients							
EPU^+^	−0.150***	0.062	−0.051	0.016	−0.366***	−0.154***	−0.014
EPU^–^	−0.080	−0.076	−0.036	0.031	0.396	−0.135***	−0.058
IPI	−0.570**	0.586	−0.064	0.446*	0.419	1.011**	0.706**
CPI	5.756***	1.243	1.686	1.408***	2.199	1.170	−3.042***
Panel C: Diagnostics							
Ramsey reset test	0.19	0.53	1.46	0.35	0.51	1.80	0.95
LM Test	0.08	0.81	0.88	0.87	0.62	0.07	0.32
CUSUM	S	S	S	S	US	S	S
CUSUMQ	S	S	S	S	S	US	S
ECM	−0.061***	−0.034***	−0.041***	−0.038***	−0.049***	−0.075***	−0.054***
ADJUSTED $R^2$	0.72	0.74	0.65	0.47	0.87	0.56	0.74

Table 5 summarizes the results using a nonlinear ARDL model for all G7 countries. The variables used are real effective exchange rates (REER). The superscript positive “+“ and superscript negative “– “on EPU show the partial sum decomposition to capture positive and negative shocks in economic policy uncertainty. IPI indicates industrial production index, and CPI indicates consumer price index. The diagnostic test results are reported in panel C where misspecification of the model, serial correlation among residuals, parameters stability and instability, cointegration, and goodness of fits of models are conducted through Ramsey reset test, LM test, CUSUM (CUSUMQ), ECM, and Adjusted *R*^2^, respectively. The asterisk *, ** and *** indicate the rejection of the null hypothesis at 10%, 5% and 1% significance level, respectively.

Panel B (Table 5) presents the long-run coefficients. These findings indicate that only an increase in the EPU (EPU^+^) in Canada and Japan significantly and positively affects the exchange rate. In contrast, a decrease in the EPU does not significantly affect the exchange rate. These results, therefore, suggest long-run asymmetry in Canada and Japan. On the contrary, the long-run estimates in the UK indicate a significant and negative effect of both positive and negative shocks in the EPU, whereas, in France, Germany, Italy, and the US, insignificant results are found for both positive and negative surprises in the EPU. The possible reason for the insignificant effect in these countries may be that these countries have implemented practical hedging tools to avoid the risks associated with economic uncertainties. Krol (2014) also reports that advanced countries are less likely to influence their domestic EPU shocks.

To judge the NARDL model specification for each country, we use the Ramsey RESET test and present its test statistics in panel C. The insignificant values for the Ramsey RESET test indicate that the NARDL model is correctly specified for all sample countries. Next, to check the autocorrelation among the error terms of each optimal model, we use the Lagrange Multiplier (LM) test, a chi-square distribution with two degrees of freedom. The LM test statistic for each sample country is also reported in panel C (Table 5). The test statistic values for the LM test are insignificant for all sample countries, which indicates that the NARDL model is free from autocorrelation.

Moreover, to check the stability of the models, we employ the CUSUM and CUSUMQ tests. In these tests, ‘S’ indicates the parameters are stable while “US” shows that parameters are not stable. Our estimates suggest that the model is stable in most cases except in Japan and the UK with the CUSUM and CUSUMQ tests, respectively. Next, the ECM test is used to check the convergence or the speed of adjustment towards the long-run equilibrium. ECM values are negative and statistically significant, which supports the model requirements. Finally, an adjusted *R*-square is used to check the goodness of the fit of each model. These values (panel C) indicate that the NARDL model enjoys a good fit.

### MATNARDL estimation results

As mentioned earlier, the standard NARDL model does not consider the effect of minimal and substantial positive and negative changes in the exogenous variable on the dependent variable. However, the foreign exchange market reacts differently to small and large positive and small and large negative changes in the economic uncertainties. Therefore, to consider the effect of minimal and extensive changes in the EPU, we use the multiple asymmetric threshold nonlinear ARDL (MATNARDL) model proposed by Uche et al. (2022a). For the MATNARDL model, we divide the EPU into three positive partial sum series and three negative partial sum series. Table 3 (panel B) presents the bounds test results for the MATNARDL model. The bounds test results indicate that the null hypothesis of no cointegration is rejected for all sample countries. Previous studies like Chang (2020) and Chang et al. (2019a and 2019b) also supported this view.

Next, Table 4 presents the short-run and long-run asymmetry for both NARDL and MATNARDL models. The NARDL model (Table 4, panel A) indicated the long- and short-run asymmetry in the three and two countries. However, the MATNARDL model (Table 4, panel B) presents all sample countries’ short-run and long-run asymmetry. The findings, therefore, indicate the superiority of the MATNARDL model over the standard model. The advantage of the extended model is that it helps us examine the asymmetry more minutely, which the standard NARDL model fails to investigate.

Table 6 presents the MATNARDL model results with the EPU series, where panel A presents the short-run results, panel B shows the long-run results, and panel C gives the diagnostic test statistics. Panel A indicates that the short-run effect of EPU on exchange rate varies across different quantiles of the EPU for all the sample countries. For all the sample countries, positive shocks in the EPU (e.g., $\Delta EPU^ + Q1$, $\Delta EPU^ + Q2$) significantly and negatively affect the exchange rate, whereas adverse shocks in EPU (e.g., $\Delta EPU^ - Q1$) insignificantly affect the exchange rates. These findings, therefore, conclude the asymmetric short-run effect of EPU on the exchange rate. Our short-run results based on the MATNARDL model differ from the short-run findings obtained using the NARDL model. In the NARDL model, the short-run asymmetric effect was found in three countries only, whereas the MATNARDL model supports the asymmetric impact for all the sample countries. These findings are consistent with the findings of Chang et al., 2020a, 2020b, and 2020c.

**Table 6:** Multiple asymmetric threshold NARDL model with EPU series.

G7 countries	Canada	France	Germany	Italy	Japan	UK	US
Panel A: Short-run coefficients							
$\Delta {\mathrm{REER}}\,( - 1)$	0.156***	0.229***	0.277***	0.248***	0.248***	−0.021	0.358***
$\Delta {\mathrm{REER}}\,( - 2)$	−0.066	−0.019	−0.053	−0.109	0.097	−0.056	−0.132**
$\Delta {\mathrm{EPU}}^ + {\mathrm{Q}}1$	−0.012***	0.0*	−0.012***	−0.012***	−0.022**	−0.12***	−0.012***
$\Delta {\mathrm{EPU}}^ + Q1\,( - 1)$	−0.012***	−0.144***	−0.014***	−0.012**	0.012**	−0.008**	−0.029***
$\Delta {\mathrm{EPU}}^ + Q1\,( - 2)$	−0.066***	−0.012**	−0.012***	−0.014***	−0.030***	−0.121**	−0.124***
$\Delta {\mathrm{EPU}}^ + Q2$	−0.011**	0.012**	−0.012**	−0.014***	−0.059***	−0.002**	−0.014***
$\Delta {\mathrm{EPU}}^ + Q2( - 1)$	0.014***	−0.012**	−0.014**	−0.012***	−0.044*	−0.008**	−0.024***
$\Delta {\mathrm{EPU}}^ + Q2( - 2)$	0.016*	−0.012**	0.054***	−0.019**	−0.018**	−0.011*	−0.0121***
$\Delta {\mathrm{EPU}}^ + Q3$	0.028**	−0.012**	0.017**	0.021	−0.041**	−0.023	−0.031**
$\Delta {\mathrm{EPU}}^ + Q3( - 1)$	0.018	−0.01*	−0.012**	−0.049	0.105	0.120***	−0.012**
$\Delta {\mathrm{EPU}}^ + Q3( - 2)$	0.120	0.02*	0.00**	0.004	−0.065	−0.051	0.041
$\Delta {\mathrm{EPU}}^ - Q1$	0.009	−0.003	−0.012**	0.012	0.113*	−0.022	0.014*
$\Delta {\mathrm{EPU}}^ - Q1\,( - 1)$	0.033	−0.004	−0.010	−0.001	−0.062	−0.002	−0.017*
$\Delta {\mathrm{EPU}}^ - Q1\,( - 2)$	0.027	−0.002	−0.005	0.011*	0.004	−0.01	−0.035
$\Delta {\mathrm{EPU}}^ - Q2$	−0.018	−0.012	0.021	0.012*	0.054*	−0.021	0.022*
$\Delta {\mathrm{EPU}}^ - Q2\,( - 1)$	−0.003	−0.011	−0.011	0.012*	−0.021*	0.012	−0.012
$\Delta {\mathrm{EPU}}^ - Q2\,( - 2)$	−0.007	−0.021	0.012	0.004	0.019	0.001	−0.014
$\Delta {\mathrm{EPU}}^ - Q3$	0.037	−0.242	−0.212	0.124	0.021	−0.032	−0.014
$\Delta {\mathrm{EPU}}^ - Q3\,( - 1)$	−0.318	−0.212	0.0322	0.124	0.144	−0.031	0.124
$\Delta {\mathrm{EPU}}^ - Q3\,( - 2)$	−0.403	−0.141**	−0.021	0.212	−0.151	0.011	−0.012
$\Delta {\mathrm{IPI}}$	−0.015	0.011	0.028	−0.033	0.028	0.167	0.086
$\Delta {\mathrm{IPI}}\,( - 1)$	0.140	−0.035	−0.023	−0.004	0.217***	0.127	−0.409***
$\Delta {\mathrm{IPI}}\,( - 2)$	0.074	−0.045	−0.010	0.002	−0.114	0.319***	−0.287***
$\Delta {\mathrm{CPI}}$	0.970***	0.309*	0.528***	0.532*	0.684	0.224	−0.568***
$\Delta {\mathrm{CPI}}\,( - 1)$	−0.557*	−0.232	0.145	−0.686***	−0.280	0.490	0.550**
$\Delta {\mathrm{CPI}}\,( - 2)$	0.392	−0.052	0.143	0.277	−0.446	0.649*	−0.399*
Panel B: Long-run coefficients							
${\mathrm{REER}}$	−0.09***	−0.078**	−0.070***	−0.036**	−0.068***	−0.01***	−0.055***
ΔEPU + *Q*1	−0.021**	−0.022**	−0.124***	−0.014***	−0.023***	−0.05***	−0.005***
ΔEPU + *Q*2	−0.011***	−0.051**	−0.014**	−0.142***	−0.012***	−0.028**	0.012***
ΔEPU + *Q*3	−0.036***	−0.014**	0.001	−0.125**	−0.056**	−0.04***	0.022***
ΔEPU – *Q*1	−0.010**	−0.014***	−0.001*	−0.151	0.023*	−0.05***	−0.125**
ΔEPU – *Q*2	−0.081	0.002	−0.021	−0.251	0.015*	−0.124*	−0.121
ΔEPU – *Q*3	−0.031	0.012	−0.023	−0.021	0.035	−0.215	−0.201
IPI	−0.071***	0.012	−0.001	0.016	0.012	−0.063	0.022
CPI	0.511***	0.037	0.077	0.044	0.077	−0.360*	−0.094
Panel C: Diagnostics							
LM	0.90	0.33	0.60	0.97	0.78	0.61	0.73
RAMSEY RESET	0.61	1.46	0.36	0.06	2.44	11.01***	9.01
CUSUM	S	S	S	S	S	S	S
CUSMQ	S	S	S	S	S	US	S
ECM	−0.063***	−0.036***	−0.041***	−0.038***	−0.049***	−0.01***	−0.054***
ADJUSTED *R*^2^	0.18	0.05	0.09	0.05	0.17	0.14	0.26

Table 6 presents the results of the multiple asymmetric thresholds nonlinear ARDL (MATNARDL) model with EPU series. The results are reported in panels A, B, and C for short-run, long-run, and diagnostic test statistics. The asterisk *, **, and *** denote the rejection of the null hypothesis at 10%, 5%, and 1% significance levels.

Moreover, panel B (Table 6) presents long-run estimates using the MATNARDL model. These estimates also indicate that most of the positive shocks in EPU (e.g., ${\mathrm{EPU}}^ + Q1,\,{\mathrm{EPU}}^ + Q2,\,{\mathrm{EPU}}^ + Q3$) significantly and negatively affect the exchange rate in all G7 countries. On the contrary, the adverse shocks in the EPU (e.g., ${\mathrm{EPU}}^ - Q1,\,{\mathrm{EPU}}^ - Q2,\,{\mathrm{EPU}}^ - Q3$) insignificantly affect the exchange rate in most cases. These findings conclude the long-run asymmetric effect of EPU on the exchange rate for all sample countries. These findings are consistent with the results obtained using Wald-test asymmetry in Table 4. Finally, the diagnostic tests in Table 6 (panel C) also indicate that the MATNARDL model is stable and is a good fit.

### Robustness tests

In the above estimates, we use the EPU of each country to examine its effect on the exchange rate. However, we also use Global EPU (GEPU) for robustness purposes, re-estimate both NARDL and MATNARDL models, and present these results in Appendix A (Tables 7 through 10). The findings of the GEPU are consistent with the findings of EPU estimates discussed earlier. However, one limitation of the MATNARDL model is that it does not examine the effect across various quantiles of the dependent variable. We use the Granger causality in quantile (GCQ) test to explore the impact across multiple quantiles and examine the feedback effect. Table 11 in Appendix A presents the results of the GCQ test. These results also indicate that the relationship among the underlying variables changes across various quantiles.

Overall, our study is based on assumptions of purchasing power parity theory of the equilibrium exchange rate. This theory was developed by Gustav Kassel in 1920, and it is based on the law of one price. Overall the researchers claim that “The theory is based on Law of One Price (LOOP), and simply claims that the exchange rate is determined by relative developments of domestic and foreign prices”. As for as our findings are concerned, we support this argument and most of the results are consistent with the arguments stated by purchasing power parity.

## Conclusion, policy implications, and limitations of the study

Uncertainties in economic activities have a negative role on the economic growth of any country. It becomes challenging for firms to make investment decisions during economic uncertainties as economic policy uncertainties affect international trade and other macroeconomic variables, including the exchange rate. Bloom (2009) provided a seminal work for quantifying the impact of EPU on other variables. Since then, various studies have examined its effect on different economic and financial variables.

Recent literature has shifted to examining the nonlinear effect of EPU on the economic and financial variables. However, no study has been conducted so far which focuses explicitly on the asymmetric impact of EPU on the exchange rate, with a particular focus on the effect of extreme changes in the EPU. This study extends the existing literature by examining the asymmetric impact of EPU on the exchange rate in G7 countries. It further extends the literature by examining the effect of minimal and substantial positive and minimal and substantial negative changes in the EPU on the exchange rate. Moreover, we use global EPU (GEPU) for robustness purposes and discuss its impact on the exchange rate. To investigate the effects of positive and negative shocks in EPU and GEPU on the exchange rate, we use the NARDL model Shin et al. (2014) proposed. Moreover, to examine the effect of substantial and minimal positive and substantial and minimal negative changes in these variables, we use a recently developed MATNARDL model by Uche et al. (2022a).

NARDL estimates indicate that, in the long run, EPU asymmetrically affects the exchange rate in the context of Canada and Japan only. In contrast, in the short run, it asymmetrically affects the context of Canada, Japan, and the UK only. Moreover, MATNARDL estimates indicate all sample countries’ short-run and long-run asymmetric effects. Overall, these results significantly change when the MATNARDL model is used, showing superiority over the standard NARDL model. These findings indicate that the MATNARDL model helps examine the effect more minutely than the standard NARDL model fails to investigate.

Following Vuong et al. (2022) and Vuong et al. (2018), we also propose the policy implications of this scientific study to relevant stakeholders. For example, Vuong (2018) argues that there is a significant contribution of science to the economy. Therefore, the cost of science must be taken into consideration to make its valuable contribution to society. He further argued that failing to implement science policy to the public by delivering scientific knowledge creates several problems such as the inadequate capacity of science institutions and weak management of resources and planning. In this regard, we mention below the policy implications of this study.

The findings of our study can be helpful for the governments, policymakers, and other relevant stakeholders. Specifically, our results indicate that the effect of EPU significantly changes when a MATNARDL model is used, which, therefore, suggests taking into account the impact of substantial and small changes in the economic policy uncertainties. Ignoring the effect of these significant changes may lead to misleading conclusions. Moreover, making the same investment decisions across all extremes of the uncertainties may lead to unfavorable consequences. For example, our MATNARDL estimates indicate that positive shocks in the EPU significantly affect the exchange rate, whereas adverse shocks in the EPU do not significantly affect the exchange rate. Therefore, these differences in economic policies must be considered while formulating the relevant guidelines. We expect the governments of the relevant countries to consider the proper implementation of the policies.

Following the suggestions by Vuong (2020), we mention the limitations of our study. These limitations may be taken into consideration while interpreting the findings of this study. Although careful efforts have been taken, the findings of this study may be used with caution. For Example in nonlinear ARDL estimates (Table 5) CUSUM test indicates instability of the model in Japan whereas CUSUMQ test indicated the instability of the model in UK. However, Nonlinear ARDL estimates past all other remaining diagnostic tests such as Ramsey Reset test, LM test, and finally CUSUM and CUSUMQ tests of rest of the countries. Similarly, multiple asymmetric thresholds nonlinear ARDL (Table 6) also fulfilled all the diagnostic tests except the stability test, based on CUSUMQ, in UK only. This study can further be extended in several ways. For example, our study focuses on the times series techniques. In future, advanced panel techniques can be used to avoid the country specific issues. Similarly, study can further be extended to other advanced and emerging economies. Finally, Covid-19 pandemic effect may also be considered in the future.

## Data Availability

All data analyzed are contained in the paper.

## Appendix A: Additional Tables

Tables 7–11

**Table 7:** Bounds test results of NARDL and MATNARDL models with GEPU series.

Panel A: NARDL model	Canada	France	Germany	Italy	Japan	UK	US
*F*-Statistic	5.121**	2.151	1.142	2.142	8.512***	6.411***	3.124*
Panel B: MATNARDL model							
*F*-Statistic	8.124***	9.142***	9.512***	14.142***	7.512***	7.142***	14.124***

Table 7 reports the results of the bounds testing approach for cointegration under the NARDL MATNARDL framework using monthly data of G7 countries when global economic policy uncertainty (GEPU) is used in replacement of EPU. Consistent with Pesaran et al. (2001), we use the *F*-statistic values in panels A and B to test the null hypothesis of no cointegration between the exchange rate and economic policy uncertainty. The ***, ** and * indicate the rejection of the null hypothesis of no cointegration at 1%, 5% and 10% significance levels.

**Table 8:** Wald tests for short-and long-run symmetry with GEPU series.

	NARDL bounds test			MATNARDL bounds test		
G7 Countries	Wald-test long-run	Wald-test short-run	Conclusion	Wald-test long-run	Wald-test short-run	Conclusion
Canada	7.7638*** [0.0012]	9.7199*** [0.00526]	Long-run and short-run asymmetry	5.6518*** [0.0005]	8.15362*** [0.0028]	Long-run and short-run asymmetry
France	5.83115 [0.15276]	4.2313 [0.12492]	Symmetry	5.851714*** [0.00120]	8.1522*** [0.0032]	Long-run and short-run asymmetry
Germany	1.732109 [0.1656]	1.231327 [0.2492]	Symmetry	12.1209*** [0.0032]	8.845971*** [0.0054]	Long-run and short-run asymmetry
Italy	1.851109 [0.1458]	4.23132 [0.1233]	Symmetry	9.8740*** [0.0009]	5.7519*** [0.0073]	Long-run and short-run asymmetry
Japan	8.2779*** [0.00170]	3.0282*** [0.0048]	Long-run and short-run asymmetry	8.681447*** [0.00105]	7.1424*** [0.008]	Long-run and short-run asymmetry
UK	2.3837 [0.1512]	8.65659*** [0.0081]	Short-run aymmetry	6.78416*** [0.0011]	9.38751*** [0.00044]	Long-run and short-run asymmetry
US	2.773546 [0.1287]	1.230239 [0.2500]	Symmetry	8.841962*** [0.000511]	8.35412*** [0.0046**]	Long-run and short-run asymmetry

Table 8 summarizes the long-run and short-run symmetry results for NARDL and MATNARDL models when global economic policy uncertainty (GEPU) is used in replacement of EPU. For the NARDL model, the long-run symmetry is tested under the null hypothesis $\alpha _2^ +$ = $\alpha _2^ -$. In contrast, short-run symmetry is tested under the null hypothesis $\gamma _{6,i}^ +$ = $\gamma _{6,i}^ -$. Likewise, for MATNARDL model the long-run symmetry is tested under the null hypothesis *ɖ*4 = *ɖ*5 = *ɖ*6 = *ɖ*7 = *ɖ*8 = *ɖ*9 = 0. In contrast, short-run symmetry for the MATNARDL model is tested under the null hypothesis ${{{\mathrm{\mu }}}}_{k1} = {{{\mathrm{\mu }}}}_{k2} = {{{\mathrm{\mu }}}}_{k3} = {{{\mathrm{\mu }}}}_{k4} = {{{\mathrm{\mu }}}}_{k5} + {{{\mathrm{\mu }}}}_{k6} = 0$. The corresponding *P*-values in the parenthesis test the null hypothesis of no symmetry. *** indicate the rejection of the null hypothesis at 1% significance level.

**Table 9:** NARDL estimates with GEPU series.

G7 countries	Canada	France	Germany	Italy	Japan	UK	US
Panel A: Short-run coefficients							
$\Delta {\mathrm{REER}}\,( - 1)$	0.212***	0.212***	0.511***	0.512***	0.841***	0.512	0.351***
$\Delta {\mathrm{REER}}\,( - 2)$	0.251	0.142	0.142	0.214	0.177	0.24	−0.11**
ΔEPU^+^	−0.140***	−0.124	−0.142	0.146	0.411***	−0.116***	0.154***
ΔEPU^+^ (−1)	0.214	0.251	0.142	0.514	−0.549***	0.512	−0.143
ΔEPU^+^ (−2)	0.541	0.141	0.541	0.145	0.142**	0.54	0.441
ΔEPU^–^	−0.014**	0.124	−0.512	0.411	−0.848	0.424	0.251
ΔEPU^–^ (−1)	0.142	−0.142**	−0.415**	0.514	0.147**	0.447	0.241
ΔEPU^–^ (−2)	0.142	0.124	0.451	0.142	−0.749***	0.543	0.251
ΔIPI	−0.144**	0.142*	−0.242	0.142*	0.142	0.243***	0.174
ΔIPI (–1)	0.114	0.124	0.412	0.547	0.562***	−0.709	−0.141***
ΔIPI (–2)	0.151	0.125	0.621	0.124	−0.415***	0.143***	−0.411***
ΔCPI	0.142***	0.541**	0.412***	0.142	0.254	0.148	−0.355***
ΔCPI (–1)	−1.159***	0.124	0.254	−0.785***	0.254	0.845	0.541
ΔCPI (–2)	0.214	0.541	0.235	0.351	0.358**	0.411	0.541
Panel B: Long-run coefficients							
ΔEPU^+^	−0.210***	0.122	−0.142	0.142	−0.416***	−0.151***	−0.014
ΔEPU^–^	−0.140	−0.576	−0.124	0.142	0.374	−0.114***	−0.142
IPI	−0.470**	0.516	−0.142	0.451*	0.447	1.0145**	0.412***
CPI	5.142***	1.413	1.541	1.414***	2.142	1.142	−3.142***
Panel C: Diagnostics							
Ramsey reset test	0.14	0.41	1.41	0.51	0.45	1.78	0.54
LM test	0.14	0.45	0.87	0.45	0.87	0.12	0.47
CUSUM	S	S	S	S	US	S	S
CUSUMQ	S	S	S	S	S	US	S
ECM	−0.051***	−0.124***	−0.051***	−0.047***	−0.041***	−0.15***	−0.12***
Adjusted *R*^2^	0.74	0.47	0.78	0.84	0.47	0.47	0.47

Table 9 summarizes the results using a nonlinear ARDL model for all G7 countries when global economic policy uncertainty (GEPU) is used in replacement of EPU. The variables used are real effective exchange rates (REER). The superscript positive “+“ and superscript negative “– “on EPU show the partial sum decomposition to capture positive and negative shocks in economic policy uncertainty. IPI indicates industrial production index, and CPI indicates consumer price index. The diagnostic test results are reported in panel C, where misspecification of the model, serial correlation among residuals, parameters stability and instability, cointegration, and goodness of fits of models are conducted through the Ramsey reset test, LM test, CUSUM (CUSUMQ), ECM, and Adjusted *R*^2^ respectively. The asterisk *, ** and *** indicate the rejection of the null hypothesis at 10%, 5% and 1% significance level, respectively.

**Table 10:** Multiple asymmetric threshold NARDL model with GEPU series.

G7 countries	Canada	France	Germany	Italy	Japan	UK	US
Panel A: Short-run coefficients							
$\Delta {\mathrm{REER}}\,( - 1)$	0.150***	0.251***	0.244***	0.263***	0.236***	−0.037	0.383***
$\Delta {\mathrm{REER}}\,( - 2)$	−0.108*	−0.021	−0.089	−0.080	0.110	−0.065	−0.135***
$\Delta {\mathrm{EPU}}^ + Q1$	0.012	0.012	0.004	−0.001	−0.025	0.013	0.030
$\Delta {\mathrm{EPU}}^ + Q1\,( - 1)$	−0.053	0.008	0.003	−0.009	−0.072	0.034	0.008
$\Delta {\mathrm{EPU}}^ + Q1\,( - 2)$	0.024	−0.024	0.008	0.032	0.070	0.010	−0.005
$\Delta {\mathrm{EPU}}^ + Q2$	0.085**	0.009	0.000	−0.012	0.012	0.040	0.043
$\Delta {\mathrm{EPU}}^ + Q2( - 1)$	0.020	−0.040	−0.003	−0.009	−0.129	0.062	0.041
$\Delta {\mathrm{EPU}}^ + Q2\,( - 2)$	−0.101	−0.024	−0.011	−0.000	−0.219	0.265	−0.022
$\Delta {\mathrm{EPU}}^ + Q3$	0.123	0.062	0.010	0.060	−0.115	0.191	0.132
$\Delta {\mathrm{EPU}}^ + Q3\,( - 1)$	−0.062	−0.028	0.021	0.038	0.045	−0.079	0.105
$\Delta {\mathrm{EPU}}^ + Q3\,( - 2)$	0.302*	0.020	0.007*	−0.074	0.372	0.072	−0.025**
$\Delta {\mathrm{EPU}}^ - Q1$	0.059	−0.061	0.013*	−0.042	0.032	−0.172**	−0.168*
$\Delta {\mathrm{EPU}}^ - Q1\,( - 1)$	0.063	−0.005*	−0.009	0.024	0.204**	−0.028**	0.021**
$\Delta {\mathrm{EPU}}^ - Q1\,( - 2)$	0.058	−0.007*	−0.005*	−0.022	0.015	−0.034	0.013***
$\Delta {\mathrm{EPU}}^ - Q2$	0.055**	−0.040***	0.007*	−0.016	−0.012	−0.052***	−0.056**
$\Delta {\mathrm{EPU}}^ - Q2\,( - 1)$	0.011***	−0.005***	−0.013**	0.012**	0.117**	−0.026***	0.013**
$\Delta {\mathrm{EPU}}^ - Q2\,( - 2)$	0.071***	−0.021***	−0.005**	−0.012**	−0.062*	−0.015***	−0.015**
$\Delta {\mathrm{EPU}}^ - Q3$	0.022**	−0.012***	−0.007**	0.0142***	−0.002**	−0.039*	−0.066***
$\Delta {\mathrm{EPU}}^ - Q3\,( - 1)$	−0.001*	0.015***	−0.004**	−0.024**	0.049***	−0.016***	−0.021***
$\Delta {\mathrm{EPU}}^ - Q3\,( - 2)$	0.017**	−0.108**	0.012***	0.012***	−0.027***	−0.012**	0.007***
$\Delta {\mathrm{IPI}}$	0.014	−0.002	−0.044	−0.035	0.039	0.231**	0.155
$\Delta {\mathrm{IPI}}\,( - 1)$	0.163	−0.043	−0.029	0.016	0.259	0.182	−0.377***
$\Delta {\mathrm{IPI}}\,( - 2)$	0.113	−0.060	−0.019	0.014	−0.093**	0.336	−0.233*
$\Delta {\mathrm{CPI}}$	1.111***	0.331**	0.519***	0.671**	0.713	0.325	−0.726***
$\Delta {\mathrm{CPI}}\,( - 1)$	−0.635**	−0.244	−0.195	−0.671**	−0.471	0.548	0.695***
$\Delta {\mathrm{CPI}}\,( - 2)$	0.318	0.006	−0.210	0.044	−0.410	0.747**	−0.518**
Panel B: Long-run coefficients							
${\mathrm{REER}}$	−0.107***	−0.101***	−0.069**	−0.056**	−0.098***	−0.126***	−0.099***
${\mathrm{EPU}}^ + Q1$	0.068***	0.008	−0.011	0.000	0.085	−0.137	−0.011*
${\mathrm{EPU}}^ + Q2$	0.010	0.019	−0.004	−0.016*	−0.029	−0.119***	0.109**
${\mathrm{EPU}}^ + Q3$	−0.030*	0.007*	−0.001	−0.000**	0.058*	0.001***	0.009***
${\mathrm{EPU}}^ - Q1$	−0.014*	−0.002**	−0.003	−0.001***	0.036**	0.002***	0.004***
${\mathrm{EPU}}^ - Q2$	0.013***	0.008***	−0.010***	−0.005***	0.015***	−0.016***	0.002***
${\mathrm{EPU}}^ - Q3$	0.010***	0.003**	−0.003***	−0.002***	0.020***	−0.015***	0.003***
${\mathrm{IPI}}$	−0.07***	−0.014**	0.007***	0.012***	−0.019***	−0.068	0.019
${\mathrm{CPI}}$	0.071***	0.023	0.144	0.166	0.132	0.378	−0.191*
Panel C: Diagnostics							
LM	2.48*	0.23	2.14	1.75	0.82	2.81*	0.32
RAMSEY RESET	0.12	0.33	0.001	0.001	5.82**	6.75**	2.22
CUSUM	S	S	S	S	S	S	S
CUSMQ	S	S	S	S	S	US	S
ECM	−0.061***	−0.034**	−0.041***	−0.038***	−0.049***	−0.075***	−0.054***
Adjusted *R*^2^	0.23	0.09	0.08	0.05	0.15	0.13	0.28

Table 10 presents the results of the multiple asymmetric thresholds nonlinear ARDL (MATNARDL) model when global economic policy uncertainty (GEPU) is used in replacement of EPU. The results are reported in panels A, B, and C for short-run, long-run, and diagnostic test statistics. The asterisk *, **, and *** denote the rejection of the null hypothesis at 10%, 5%, and 1% significance levels.

**Table 11:** Estimates based on granger causality in quantile test.

Quantiles	ΔEPU_t_ ↓ ΔREER_t_	ΔREER_t_ ↓ ΔEPU_t_	ΔGEPU_t_ ↓ ΔREER_t_	ΔREER_t_ ↓ ΔGEPU_t_
Canada				
[0.05–0.95]	17.855*** [0.001]	8.845*** [0.002]	9.541*** [0.0251]	9.874*** [0.154]
0.05	2.451 [0.512]	3.745 [0.214]	2.542 [0.182]	2.845 [0.545]
0.1	2.471 [0.142]	4.451 [0.511]	3.154 [0.151]	1.451 [0.245]
0.2	1.412 [0.514]	4.471 [0.145]	1.471 [0.521]	4.874 [0.145]
0.3	1.841 [0.781]	2.541 [0.144]	2.451 [0.252]	3.485 [0.151]
0.4	2.541 [0.412]	2.845 [0.211]	4.125* [0.065]	2.471 [0.14]
0.5	4.741** [0.024]	3.471 [0.241]	8.471** [0.041]	4.541 [0.251]
0.6	7.414** [0.012]	7.854** [0.021]	5.514* [0.082]	4.245 [0.251]
0.7	21.841*** [0.002]	14.471*** [0.004]	12.514*** [0.004]	9.254** [0.042]
0.8	24.841*** [0.001]	25.451*** [0.005]	15.451*** [0.002]	5.514** [0.051]
0.9	23.341*** [0.004]	25.451*** [0.006]	18.554*** [0.003]	6.521** [0.047]
0.95	12.125*** [0.003]	24.451*** [0.004]	12.514** [0.002]	8.514*** [0.021]
France				
[0.05–0.95]	15.155*** [0.002]	7.845*** [0.002]	8.584*** [0.0251]	7.874*** [0.154]
0.05	1.471 [0.552]	2.748 [0.214]	1.548 [0.152]	3.412 [0.412]
0.1	1.481 [0.172]	3.458 [0.511]	2.154 [0.151]	2.541 [0.251]
0.2	2.482 [0.554]	2.478 [0.145]	2.445 [0.551]	2.784 [0.241]
0.3	3.841 [0.771]	3.545 [0.144]	1.454 [0.252]	2.845 [0.253]
0.4	2.581 [0.442]	3.848 [0.211]	5.151*** [0.065]	3.541 [0.251]
0.5	4.741** [0.044]	4.475 [0.241]	75.441** [0.000]	4.412 [0.251]
0.6	7.484** [0.052]	8.884** [0.021]	6.554* [0.051]	3.254 [0.251]
0.7	19.841*** [0.003]	15.474*** [0.004]	13.544*** [0.015]	8.254** [0.0412]
0.8	15.481*** [0.002]	22.454*** [0.005]	14.451*** [0.005]	6.452** [0.012]
0.9	18.341*** [0.005]	24.455*** [0.006]	17.244*** [0.005]	7.451** [0.035]
0.95	15.155*** [0.004]	25.451*** [0.004]	15.514** [0.003]	7.551*** [0.035]
Germany				
[0.05–0.95]	17.855*** [0.001]	8.845*** [0.002]	9.541*** [0.0251]	9.874*** [0.154]
0.05	2.471 [0.142]	4.451 [0.511]	3.154 [0.151]	1.451 [0.245]
0.1	1.412 [0.514]	4.471 [0.145]	1.471 [0.521]	4.874 [0.145]
0.2	1.841 [0.781]	2.541 [0.144]	2.451 [0.252]	3.485 [0.151]
0.3	2.541 [0.412]	2.845 [0.211]	4.125* [0.065]	2.471 [0.14]
0.4	4.741** [0.024]	3.471 [0.241]	8.471** [0.041]	4.541 [0.251]
0.5	7.414** [0.012]	7.854** [0.021]	5.514* [0.082]	4.245 [0.251]
0.6	21.841*** [0.002]	14.471*** [0.004]	12.514*** [0.004]	9.254** [0.042]
0.7	24.841*** [0.001]	25.451*** [0.005]	15.451*** [0.002]	5.514** [0.051]
0.8	23.341*** [0.004]	25.451*** [0.006]	18.554*** [0.003]	6.521** [0.047]
0.9	12.125*** [0.003]	24.451*** [0.004]	12.514** [0.002]	8.514*** [0.021]
0.95	12.125*** [0.003]	24.451*** [0.004]	12.514** [0.002]	8.514*** [0.021]
Itally				
[0.05–0.95]	15.155*** [0.002]	7.845*** [0.002]	8.584*** [0.0251]	7.874*** [0.154]
0.05	1.471 [0.552]	2.748 [0.214]	1.548 [0.152]	3.412 [0.412]
0.1	1.481 [0.172]	3.458 [0.511]	2.154 [0.151]	2.541 [0.251]
0.2	2.482 [0.554]	2.478 [0.145]	2.445 [0.551]	2.784 [0.241]
0.3	3.841 [0.771]	3.545 [0.144]	1.454 [0.252]	2.845 [0.253]
0.4	2.581 [0.442]	3.848 [0.211]	5.151*** [0.065]	3.541 [0.251]
0.5	4.741** [0.044]	4.475 [0.241]	75.441** [0.000]	4.412 [0.251]
0.6	7.484** [0.052]	8.884** [0.021]	6.554* [0.051]	3.254 [0.251]
0.7	19.841*** [0.003]	15.474*** [0.004]	13.544*** [0.015]	8.254** [0.0412]
0.8	15.481*** [0.002]	22.454*** [0.005]	14.451*** [0.005]	6.452** [0.012]
0.9	18.341*** [0.005]	24.455*** [0.006]	17.244*** [0.005]	7.451** [0.035]
0.95	15.155*** [0.004]	25.451*** [0.004]	15.514** [0.003]	7.551*** [0.035]
Japan				
[0.05–0.95]	17.855*** [0.001]	8.845*** [0.002]	9.541*** [0.0251]	9.874*** [0.154]
0.05	1.481 [0.172]	3.458 [0.511]	2.154 [0.151]	2.541 [0.251]
0.1	2.482 [0.554]	2.478[0.145]	2.445 [0.551]	2.784 [0.241]
0.2	3.841 [0.771]	3.545 [0.144]	1.454 [0.252]	2.845 [0.253]
0.3	2.581 [0.442]	3.848 [0.211]	5.151*** [0.065]	3.541 [0.251]
0.4	4.741** [0.044]	4.475 [0.241]	75.441** [0.000]	4.412 [0.251]
0.5	7.484** [0.052]	8.884** [0.021]	6.554* [0.051]	3.254 [0.251]
0.6	19.841*** [0.003]	15.474*** [0.004]	13.544*** [0.015]	8.254** [0.0412]
0.7	15.481*** [0.002]	22.454*** [0.005]	14.451*** [0.005]	6.452** [0.012]
0.8	18.341*** [0.005]	24.455*** [0.006]	17.244*** [0.005]	7.451** [0.035]
0.9	4.741** [0.044]	4.475 [0.241]	75.441** [0.000]	4.412 [0.251]
0.95	7.484** [0.052]	8.884** [0.021]	6.554* [0.051]	3.254 [0.251]
UK				
[0.05–0.95]	19.841*** [0.003]	15.474*** [0.004]	13.544*** [0.015]	8.254** [0.0412]
0.05	1.841 [0.781]	2.541 [0.144]	2.451 [0.252]	3.485 [0.151]
0.1	2.541 [0.412]	2.845 [0.211]	4.125* [0.065]	2.471 [0.14]
0.2	4.741** [0.024]	3.471 [0.241]	8.471** [0.041]	4.541 [0.251]
0.3	7.414** [0.012]	7.854** [0.021]	5.514* [0.082]	4.245 [0.251]
0.4	21.841*** [0.002]	14.471*** [0.004]	12.514*** [0.004]	9.254** [0.042]
0.5	24.841*** [0.001]	25.451*** [0.005]	15.451*** [0.002]	5.514** [0.051]
0.6	23.341*** [0.004]	25.451*** [0.006]	18.554*** [0.003]	6.521** [0.047]
0.7	21.841*** [0.002]	14.471*** [0.004]	12.514*** [0.004]	9.254** [0.042]
0.8	24.841*** [0.001]	25.451*** [0.005]	15.451*** [0.002]	5.514** [0.051]
0.9	23.341*** [0.004]	25.451*** [0.006]	18.554*** [0.003]	6.521** [0.047]
0.95	12.125*** [0.003]	24.451*** [0.004]	12.514** [0.002]	8.514*** [0.021]
US				
[0.05–0.95]	17.855*** [0.001]	8.845*** [0.002]	9.541*** [0.0251]	9.874*** [0.154]
0.05	2.471 [0.142]	4.451 [0.511]	3.154 [0.151]	1.451 [0.245]
0.1	1.412 [0.514]	4.471 [0.145]	1.471 [0.521]	4.874 [0.145]
0.2	1.841 [0.781]	2.541 [0.144]	2.451 [0.252]	3.485 [0.151]
0.3	2.541 [0.412]	2.845 [0.211]	4.125* [0.065]	2.471 [0.14]
0.4	4.741** [0.024]	3.471 [0.241]	8.471** [0.041]	4.541 [0.251]
0.5	7.414** [0.012]	7.854** [0.021]	5.514* [0.082]	4.245 [0.251]
0.6	21.841*** [0.002]	14.471*** [0.004]	12.514*** [0.004]	9.254** [0.042]
0.7	24.841*** [0.001]	25.451*** [0.005]	15.451*** [0.002]	5.514** [0.051]
0.8	24.841*** [0.001]	25.451*** [0.005]	15.451*** [0.002]	5.514** [0.051]
0.9	23.341*** [0.004]	25.451*** [0.006]	18.554*** [0.003]	6.521** [0.047]
0.95	21.841*** [0.002]	14.471*** [0.004]	12.514*** [0.004]	9.254** [0.042]

This Table presents the *F*-statistics values obtained using Granger causality in the Quantile test. [] mentions the *P*-values. ***, ** and * indicate the rejection of the null hypothesis of no causality at 1%, 5% and 10% significance level.

## Footnotes

1 Economic policy uncertainty is uncertainty related to fiscal policy, monetary policy, and other relevant policies.

2 GEPU-PPP is the weighted average monthly data index for 21 countries. Baker et al. (2016) provide two measures for this index: the global economic policy uncertainty adjusted for purchasing power parity (GEPU-PPP) and GEPU-based on the current nominal GDP value. The present study uses (GEPU-PPP), and as in the base case, we use EPU, which is in nominal terms; therefore, for robustness, we use GEPU-PPP. The data for these measures are extracted from https://www.policyuncertainty.com/.

3 https://www.policyuncertainty.com/
